# Characterization of gene expression profiles for different types of mast cells pooled from mouse stomach subregions by an RNA amplification method

**DOI:** 10.1186/1471-2164-10-35

**Published:** 2009-01-20

**Authors:** Soken Tsuchiya, Yuki Tachida, Eri Segi-Nishida, Yasushi Okuno, Shigero Tamba, Gozoh Tsujimoto, Satoshi Tanaka, Yukihiko Sugimoto

**Affiliations:** 1 Department of Physiological Chemistry, Graduate School of Pharmaceutical Sciences, Kyoto University, Sakyo-ku, Kyoto 606-8501, Japan; 2 Department of Systems Bioscience for Drug Discovery, Graduate School of Pharmaceutical Sciences, Kyoto University, Sakyo-ku, Kyoto 606-8501, Japan; 3 Department of PharmacoInformatics, Graduate School of Pharmaceutical Sciences, Kyoto University, Sakyo-ku, Kyoto 606-8501, Japan; 4 Department of Genomic Drug Discovery Science, Graduate School of Pharmaceutical Sciences, Kyoto University, Sakyo-ku, Kyoto 606-8501, Japan; 5 Department of Immunobiology, School of Pharmacy and Pharmaceutical Sciences, Mukogawa Women's University, Nishinomiya, Hyogo 663-8179, Japan

## Abstract

**Background:**

Mast cells (MCs) play pivotal roles in allergy and innate immunity and consist of heterogenous subclasses. However, the molecular basis determining the different characteristics of these multiple MC subclasses remains unclear.

**Results:**

To approach this, we developed a method of RNA extraction/amplification for intact *in vivo *MCs pooled from frozen tissue sections, which enabled us to obtain the global gene expression pattern of pooled MCs belonging to the same subclass. MCs were isolated from the submucosa (sMCs) and mucosa (mMCs) of mouse stomach sections, respectively, 15 cells were pooled, and their RNA was extracted, amplified and subjected to microarray analysis. Known marker genes specific for mMCs and sMCs showed expected expression trends, indicating accuracy of the analysis.

We identified 1,272 genes showing significantly different expression levels between sMCs and mMCs, and classified them into clusters on the basis of similarity of their expression profiles compared with bone marrow-derived MCs, which are the cultured MCs with so-called 'immature' properties. Among them, we found that several key genes such as *Notch4 *had sMC-biased expression and *Ptgr1 *had mMC-biased expression. Furthermore, there is a difference in the expression of several genes including extracellular matrix protein components, adhesion molecules, and cytoskeletal proteins between the two MC subclasses, which may reflect functional adaptation of each MC to the mucosal or submucosal environment in the stomach.

**Conclusion:**

By using the method of RNA amplification from pooled intact MCs, we characterized the distinct gene expression profiles of sMCs and mMCs in the mouse stomach. Our findings offer insight into possible unidentified properties specific for each MC subclass.

## Background

Mast cells (MCs) are derived from hematopoietic stem cells and play important roles in allergic responses, innate immunity and defense against parasite infection. Unlike other blood cells, MCs migrate into peripheral tissues as immature progenitors and differentiate into mature mast cells. One of the unique features of MCs is that they show a variety of phenotypes depending on the different tissue microenvironment of their maturation [1]. In MCs, various MC-specific serine proteases are stored in the secretory granules, and their gene and protein expressions are dramatically altered when their cell environment is altered. For example, Reynolds *et al. *have shown that at least six distinct members of mouse MC-specific serine proteases are expressed in different combinations in different mast cell populations [2]. In addition, recent studies have shown that mature MCs vary in terms of what surface receptors and lipid mediators they express [3,4]. Because each mast cell population *in vivo *must play a specific role in the body, it is important to determine the character of each population of MCs.

Comprehensive gene expression analysis is a powerful approach to understand the characterization of various MC subpopulations. To date, several studies on microarray analysis of MCs have been conducted [5-7], but most of them dealt with MCs cultured *in vitro*. Alternatively, gene expression profiles of MCs isolated from skin and lung have been analyzed [3,8-10]. However, the numbers of MCs analyzed as one sample were relatively high and they were exposed to physical forces, enzymes and the anti-Kit antibody for purification, during which the original properties of the MCs may have been affected.

In the gastrointestinal tract, there are MCs that are mainly classified into two subclasses; mucosal MCs (mMCs) and submucosal MCs (sMCs) on the basis of their location, morphology (size and shape) and granule contents [11,12]. mMCs are mainly found in the mucosa of the gastrointestinal system, having chondroitin sulfate-containing granules, which are stained with toluidine blue but not safranin, and their activation occurs during parasite infection [13], while sMCs are localized in the submucosa of the gastrointestinal tract and their granules are rich in heparin and stained with both toluidine blue and safranin [1,11]. However, the molecular basis determining the differences in biochemical properties of these two MC subclasses remains uncertain, partially due to the difficulty of their isolation.

To overcome these problems, here we established a method of RNA amplification from intact MCs isolated from frozen tissue sections, which enables us to conveniently obtain the global gene expression pattern of MCs in various tissues. To validate this method, we first determined the minimum cell number required to achieve reproducible RNA amplification. We then compared the gene expression profiles obtained from small numbers of mMCs and sMCs in the mouse stomach, and found several key genes to be specifically expressed in one subclass of MCs, which may reflect some aspects of the distinct properties between the two MC subclasses in the gastrointestinal tract.

## Results and discussion

### Development of an RNA amplification protocol to obtain gene expression profiles from a small amount of RNA

**Figure 1:**
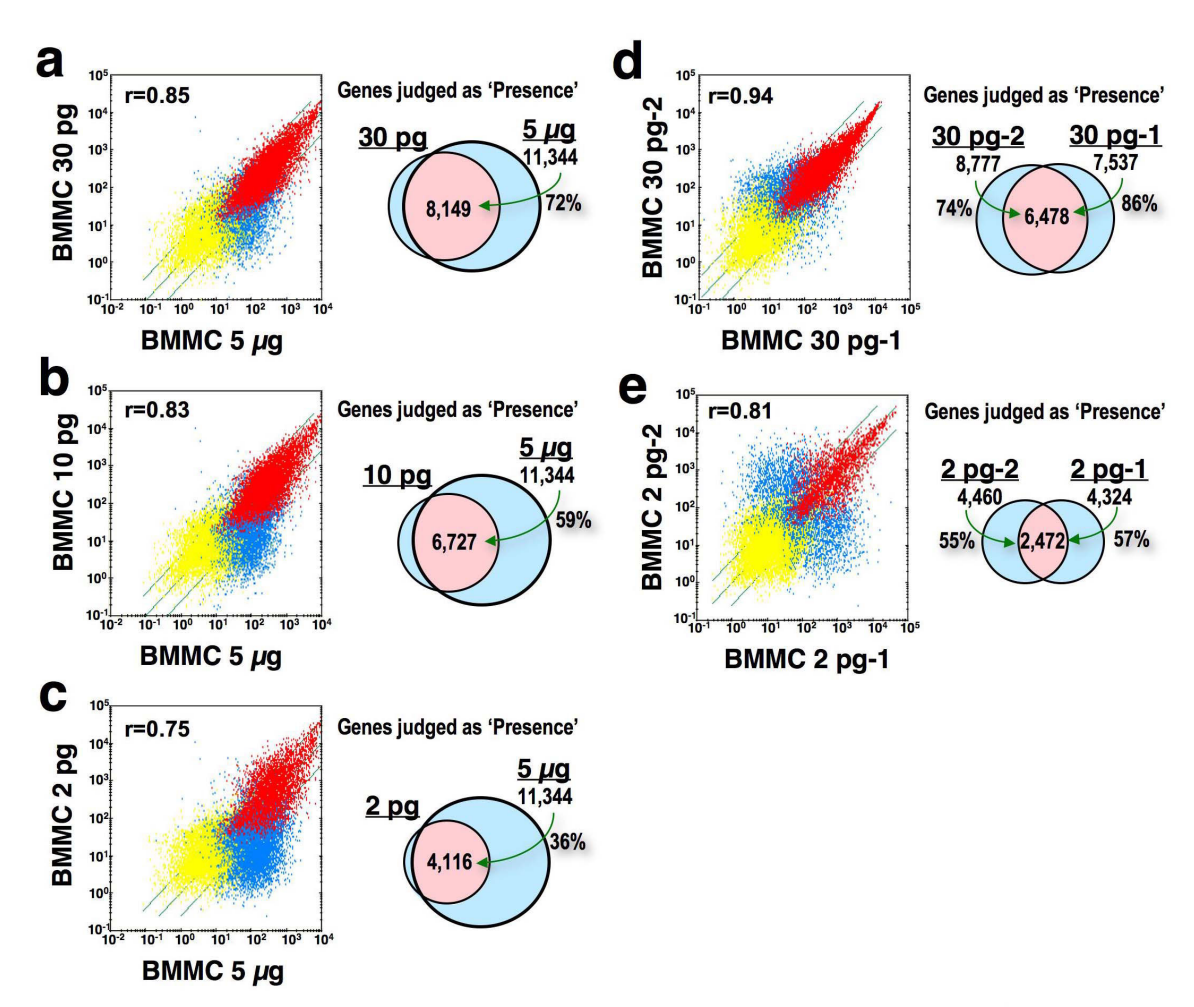
**Comparisons of three round-amplified products starting with very small quantities of RNA**. (**a-c**) Amplification biases in the products starting from a small quantity of RNA. Scatter plots of signal intensity obtained from 5 μg of BMMC RNA prepared by the standard protocol and from 30 pg (*a*), 10 pg (*b*) and 2 pg (*c*) of BMMC RNA prepared by three rounds of amplification are shown. (**d, e**) Reproducibility of the three-round amplification of a small quantity of RNA. Scatter plots of signal intensity between two independent products from 30 pg of BMMC RNA (BMMC 30 pg-1 and BMMC 30 pg-2) (*d*) or from 2 pg of BMMC RNA (BMMC 2 pg-1 and BMMC 2 pg-2) (*e*), are shown. Red dots show probe sets judged as "Presence", and yellow dots represent probe sets judged as "Absence" in both arrays. Blue dots show probe sets judged as "Presence" only in either array. The correlation coefficients (r) are presented. The same, four-fold induction and suppression thresholds are indicated as diagonal lines. Genes judged as "Presence" are placed in groups corresponding to pairwise overlaps shown in the accompanying Venn diagrams.

To gain insight into the functional differences between the different subclasses of MCs, we employed three rounds of the T7-based RNA amplification method. Based on the preliminary experiments using peritoneal MCs and bone marrow-derived MCs (BMMCs), we estimated that a single MC yields 2 pg of RNA. Before we performed comparative analysis of MCs from different tissues, we first evaluated the accuracy and reproducibility of three rounds of the T7-based RNA amplification method, starting with the amount of RNA that can be obtained from a single MC. To assess this, we first compared the microarray results obtained from 5 μg of BMMC RNA prepared by the standard protocol with those obtained from the same RNA diluted 10^5^- or 10^6^-fold (30 pg, 10 pg and 2 pg) and subjected to three rounds of T7-based amplification (Figure 1a–c). Although three rounds of amplification yielded enough quantity of RNA for microarray analysis (>20 μg) even in the case of 2 pg RNA, scatter plot analysis revealed that the qualities of the obtained results were quite different between the samples from 5 μg and 2 pg RNA. The genes judged as 'Presence' in both 30 pg and 5 μg of RNA were 8,149 genes, which corresponded to 72% of genes judged as 'Presence' in the 5 μg of RNA (11,344 genes; Figure 1a), while only 4,116 genes were judged as 'Presence' in both 2 pg and 5 μg of RNA, which corresponded to only 36% of genes judged as 'Presence' in the 5 μg RNA (Figure 1c). The decrease in the number of genes judged as 'Presence' in the diluted samples (30 pg, 10 pg and 2 pg) may be due to the loss of low copy number RNA species during amplification.

**Figure 2:**
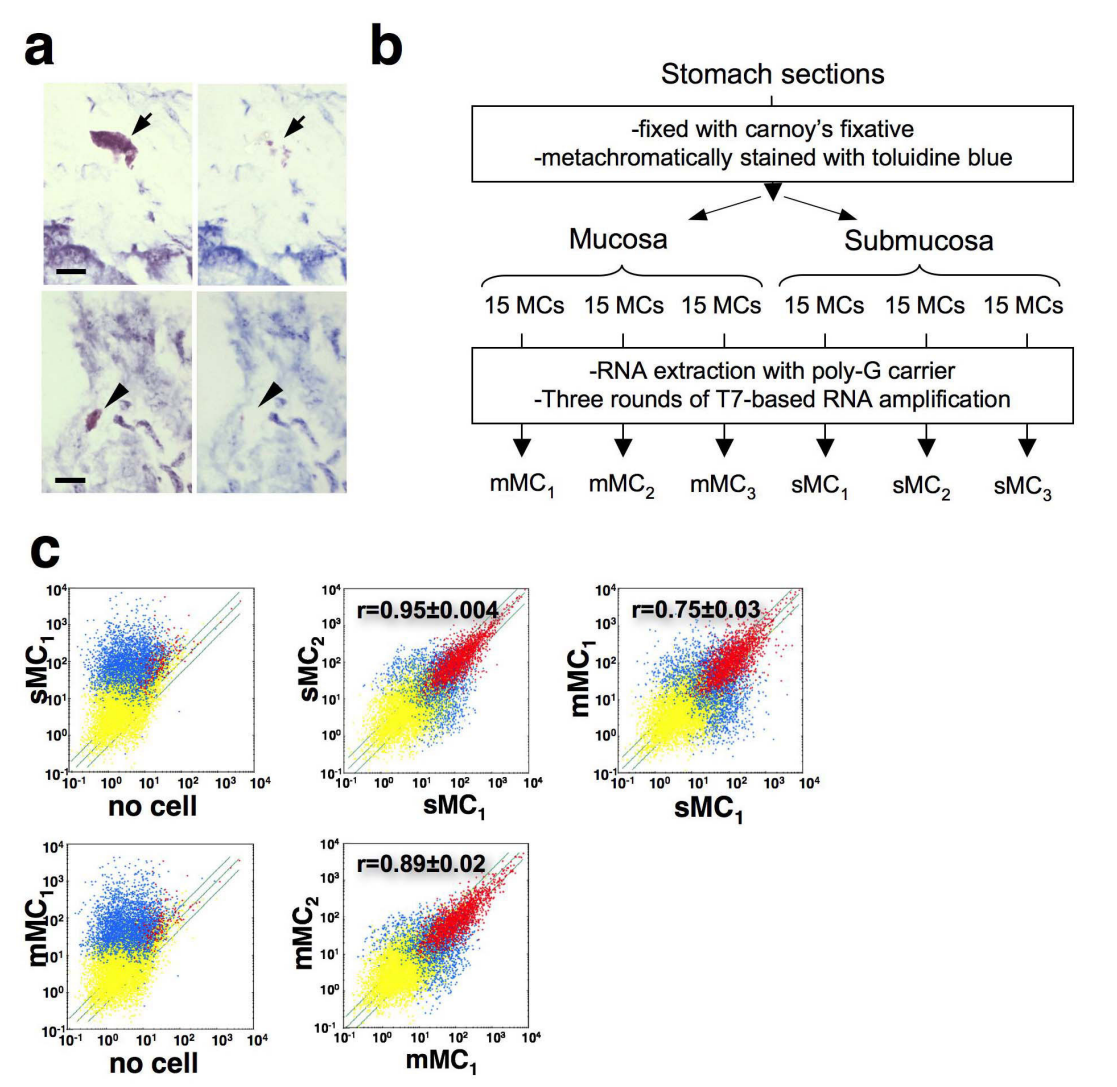
**Gene expression profiles of sMCs and mMCs from stomach tissue**. (**a**) Isolation of toluidine blue-stained MCs in the submucosa (sMC; *upper panels*) and the mucosa (mMC; *lower panels*) of stomach sections. A sMC (*arrow*) and mMC (*arrowhead*) that was metachromatically stained with toluidine blue before microdissection (*left panels*) disappeared after microdissection with a patch pipette (*right panels*). *Bars*, 10 μm. (**b**) Outline of the experimental strategy. (**c**) Labeled and fragmented antisense RNAs of three individual sMC samples, three individual mMC samples and the 'no cell' samples were hybridized to a Murine Array. Scatter plots for 'no cell' (x axis) and sMC_1 _(y axis) (*upper left*), 'no cell' (x axis) and mMC_1 _(y axis) (*lower left*), sMC_1 _(x axis) and sMC_2 _(y axis) (*upper center*), mMC_1 _(x axis) and mMC_2 _(y axis) (*lower center*), sMC_1 _(x axis) and mMC_1 _(y axis) (*upper right*) are shown. The correlation coefficients (r) for comparison within sMC_1–3_, within mMC_1–3 _and between sMCs and mMCs are presented as means ± S.D. Red dots show probe sets judged as "Presence", and yellow dots represent probe sets judged as "Absence" in both arrays. Blue dots show probe sets judged as "Presence" only in either array. The same, two-fold induction and suppression thresholds are indicated as diagonal lines.

We next examined the reproducibility of the microarray results obtained from two sets of 30 pg BMMC RNA samples (30 pg-1 and 30 pg-2) or two sets of 2 pg samples (2 pg-1 and 2 pg-2) (Figure 1d and 1e). In the 30 pg RNA samples, 7,537 (30 pg-1) and 8,777 (30 pg-2) genes were judged as 'Presence'. However, only 4,324 (2 pg-1) and 4,460 (2 pg-2) genes were judged as 'Presence' in each 2 pg RNA sample, again suggesting the loss of low copy number RNAs during amplification from a small amount of RNA. As to the reproducibility, 86% of the 'Presence' genes in the 30 pg-1 and 74% of 'Presence' genes in the 30 pg-2 sample were judged as 'Presence' in both 30 pg RNA samples, while only 57% of 'Presence' genes in the 2 pg-1 and 55% of 'Presence' genes in the 2 pg-2 sample were judged as 'Presence' in both 2 pg RNA samples. These results suggested that the amplified products from the RNA from a single MC (about 2 pg) by the current method may include considerable amplification artifacts causing problems in accuracy and reproducibility. On the other hand, because of the higher reproducibility (>74%), we concluded that amplification from 30 pg RNA collected from 15 MCs would be suitable for the practical analysis of tissue MCs. Based on these results, we set our goal in this study to acquire gene expression profiles of MCs pooled from different regions. To minimize the influence of cell-to-cell variations within the same class and potential amplification artifacts, we prepared three sets of 15 MCs for each region and compared genes with significantly different expression between MCs from the different regions (Figure 2b). We chose stomach as the source organ, since we can isolate two kinds of MCs from the mucosa (mMC) and the submucosa (sMC) regions of the same sections, and mMCs and sMCs have been suspected to be different in several MC properties such as protease expression profile and sensitivity to safranin staining [1,11].

### Gene expression profiles of submucosal and mucosal MCs from the stomach

To visualize two kinds of MCs in the stomach without causing RNA degradation, the sections were fixed with carnoy's fixative and metachromatically stained with toluidine blue for a few seconds. sMCs and mMCs were microdissected using a patch pipette (Figure 2a and 2b). We prepared three sets of 15 MCs for each region, extracted their RNA and individually amplified them (sMC_1_, sMC_2_, sMC_3_, and mMC_1_, mMC_2_, mMC_3_). To improve the recovery of the extraction of as little as 30 pg of RNA, we used 'poly G' as a carrier, which does not interfere with the following RNA amplification or hybridization of the amplified product to the array (data not shown). To examine the effects of nonspecifically amplified artifact products, we performed the RNA extraction/amplification procedure without adding microdissected cells ("no cell") as a negative control (described in "*Materials and methods*"). The amplified RNAs of sMCs, mMCs and the "no cell" control were separately hybridized to a murine microarray. The signal values in the "no cell" sample were low in general and similar to the background levels (Figure 2c). The scatter plots of the samples independently prepared within the same group (e.g. sMC_1 _vs sMC_2_) showed a similar expression pattern; the average correlation coefficient for all probe-sets was 0.945 ± 0.004 and 0.893 ± 0.019 in sMCs and mMCs, respectively (*n *= 3). In contrast, the average correlation coefficient between sMCs and mMCs was 0.752 ± 0.034 (*n *= 3), which was much lower than those within the same group, suggesting that their gene expression patterns are different.

**Figure 3:**
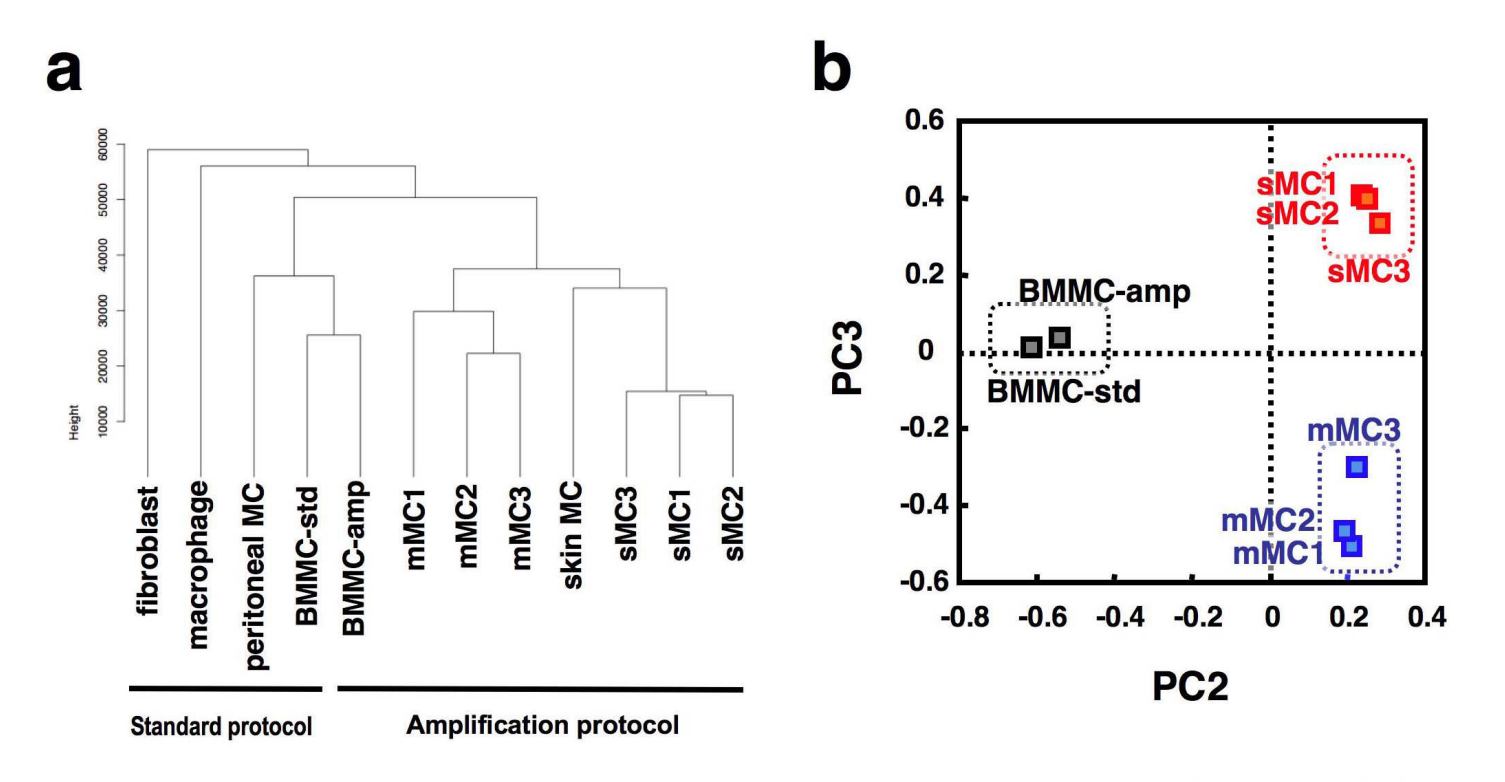
**Global gene expression analysis of sMC_1–3 _and mMC_1–3_**. (**a**) Hierarchical clustering of global gene expression of various preparations of MCs and non-MCs. Three-round amplified products of sMC_1–3_, mMC_1–3_, skin MCs and BMMCs, and the standard products of BMMCs, peritoneal MCs, macrophages and fibroblasts were analyzed. (**b**) The principal component analysis (PCA) reveals different gene expression profiles of sMC_1–3_, mMC_1–3_, and two preparations of BMMCs. The blue dotted square indicates mMCs, the red dotted square indicates sMCs, and the black dotted square indicates BMMCs.

We further evaluated the accuracy and reproducibility of our method by other comprehensive analyses (hierarchical clustering analysis and principal component analysis [PCA]) using all probe sets. Microarray data obtained from sMCs, mMCs, skin-derived MCs, peritoneal MCs, BMMCs and non-MCs (macrophages and fibroblasts) were applied to these analyses. We first checked whether the amplification process in our method affects the global expression profile due to non-linear amplification. The results from the BMMC samples using RNA prepared by the standard protocol (BMMC-std) or the amplification method (BMMC-amp) were subjected to these analyses. Both hierarchical clustering analysis and PCA revealed that microarray data from BMMC-std and BMMC-amp were clustered in the same group (Figure 3a and 3b), suggesting that the global similarity in gene expression profiles is maintained during the amplification process. We next examined the similarity of expression patterns in three independent sMC or mMC samples. Upon clustering analysis and PCA, sMC_1–3 _and mMC_1–3 _were clustered in the same group, respectively. PCA also showed that the expression profiles of sMCs, mMCs and BMMCs are mutually different (Figure 3b).

We then compared the stomach-derived MCs (sMCs and mMCs) with skin-derived MCs, peritoneal MCs, BMMCs and non-MCs (macrophages and fibroblasts) by clustering analysis. The tissue-derived MCs (stomach MCs and skin MCs) were clustered separately from peritoneal MCs and BMMCs. These results may reflect different properties between tissue-derived MCs with firm adhesion to the neighboring cells and floating MCs without a tight contact. As to the similarity of MCs with fibroblasts and macrophages, it is reasonable that fibroblasts are most distant from MCs and macrophages are closer to MCs as a leukocyte family.

### Validation of microarray results by real time RT-PCR analysis

**Figure 4:**
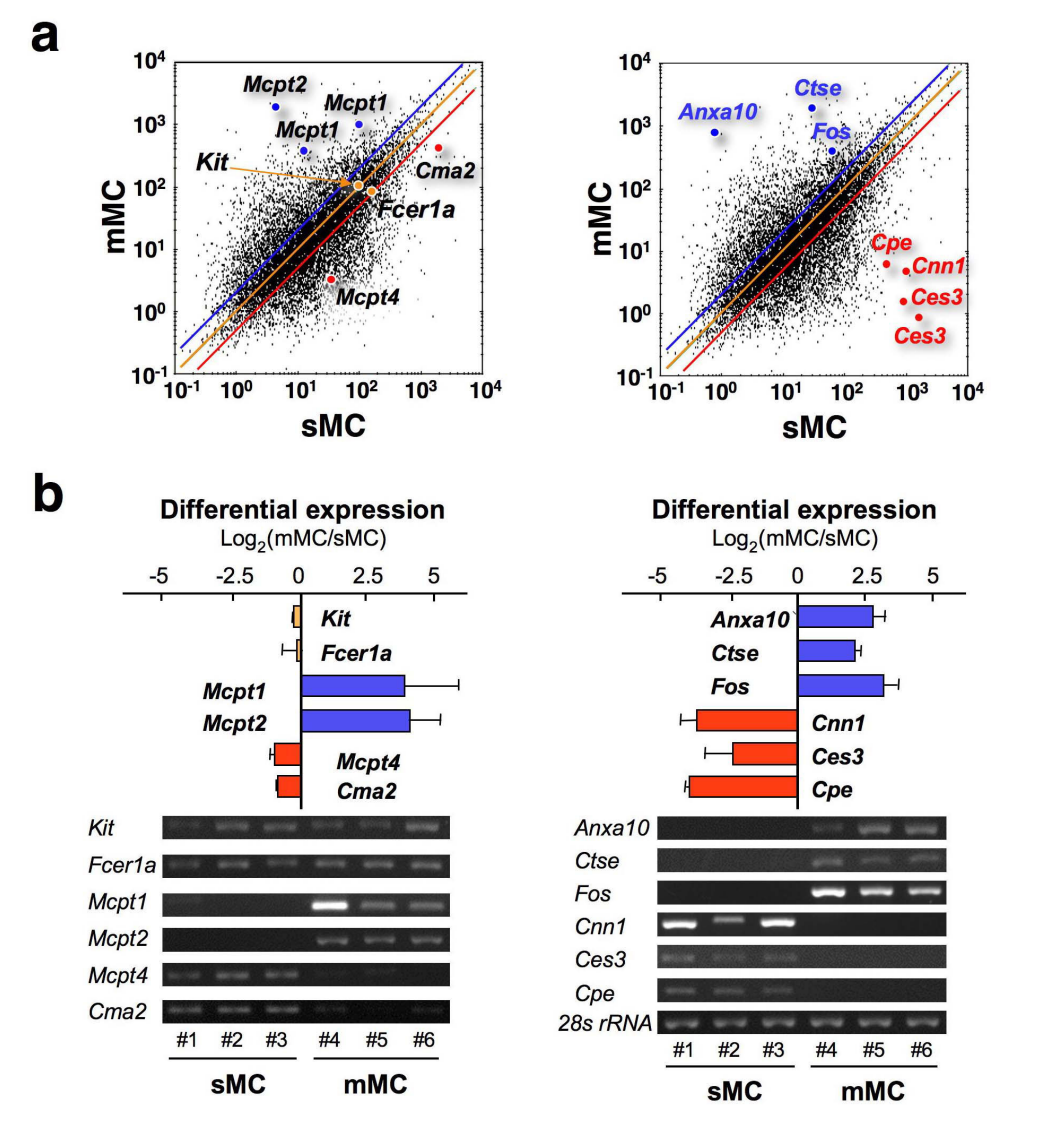
**Validation of the differentially expressed genes between sMCs and mMCs**. (**a**) sMC-specific (*Cma2*, *Mcpt4*), mMC-specific (*Mcpt1*, *Mcpt2*) and MC-common markers (*Fcer1a *and *Kit*) (*left panel*) and six randomly selected genes (*Ces3*, *Cnn1*, *Cpe*, *Anxa10*, *Ctse *and *Fos*) (*right panel*) are indicated in the representative scatter correlation graphs between sMC_1 _and mMC_1_. The same, two-fold induction and suppression thresholds are indicated as a yellow, blue and red line, respectively. (**b**) The expression levels of the genes in (a) were verified by real-time RT-PCR. The values represent the ratio of relative expression levels of mMCs to sMCs, and are shown as mean ± S.D. (n = 3). The specificity of the PCR product was confirmed by gel electrophoresis and analysis of the melting temperature. The expression level of each gene was normalized to 28S ribosomal RNA.

We next investigated whether the hybridization signals of known marker genes specific for sMCs and mMCs showed the expected expression trends [12,14]. The mMC-specific genes, mast cell protease 1 (*Mcpt1*) and 2 (*Mcpt2*) showed higher values in mMCs, while the sMC-specific marker genes, mast cell protease 4 (*Mcpt4*) and chymase 2 (*Cma2*), showed higher signal values in sMCs (Table 1 and Figure 4a) [15-29]. On the other hand, MC-common markers such as kit oncogene (*Kit*) and Fcε receptor (*Fcer1a*) showed significant signal values with no bias between mMCs and sMCs. To further evaluate the results, we measured the expression levels of these marker genes by real-time RT-PCR using RNA from the independently isolated MCs (Figure 4b). Moreover, we randomly selected three genes showing 'mMC-biased' expression and another three genes showing 'sMC-biased' expression; expression of these genes in MCs has not been reported previously (Figure 4a). There were no significant differences in the expression levels of *Kit *and *Fcer1a *between mMCs and sMCs. In contrast, the mMC-specific markers *Mcpt1 *and *Mcpt2 *and the 'mMC-biased' genes, *Anxa10, Ctse*, and *Fos *showed higher expression in mMCs, and the sMC-specific markers *Mcpt4 *and *Cma2 *and the 'sMC-biased' genes, *Cnn1, Ces3*, and *Cpe *showed higher expression in sMCs. These results indicate that the microarray results are reliable and reflect the gene expression profiles of intact sMCs and mMCs in the stomach.

**Table 1:** Summary of genes examined by real-time PCR analysis.

Gene Symbol	Gene Name	RefSeq Transcript ID	Reference
*Kit*	kit oncogene	NM_021099	15
*Fcer1a*	Fc fragment of IgE, high affinity I, receptor for α polypeptide	NM_010184	16
*Mcpt1*	mast cell protease 1	NM_008570	17, 18
*Mcpt2*	mast cell protease 2	NM_008571	19
*Mcpt4*	mast cell protease 4	NM_010779	2, 20
*Cma2*	chymase 2, mast cell (mast cell protease 10)	NM_001024714	14*
*Anxa10*	annexin A10	NM_011922	21
*Ctse*	cathepsin E	NM_007799	22
*Fos*	FBJ osteosarcoma oncogene	NM_010234	23
*Ptgr1*	Prostaglandin reductase 1 (leukotriene B_4 _12-hydroxydehydrogenase)	NM_025968	24 (porcine)
*Cnn1*	calponin 1	NM_009922	25
*Ces3*	carboxylesterase 3	NM_053200	26
*Cpe*	carboxypeptidase E	NM_013494	27 (bovine)
*Notch4*	Notch gene homolog 4	NM_010929	28
28S rRNA	28S ribosomal RNA	NR_003279	29

*, The coding sequence presented in this paper is the N-terminus truncated-form of *Cma2*, while the RefSeq "NM_001024714" is the complete sequence of *Cma2*.

### Clustering analysis of the gene expression profiles and functional categorization between sMCs and mMCs

**Figure 5:**
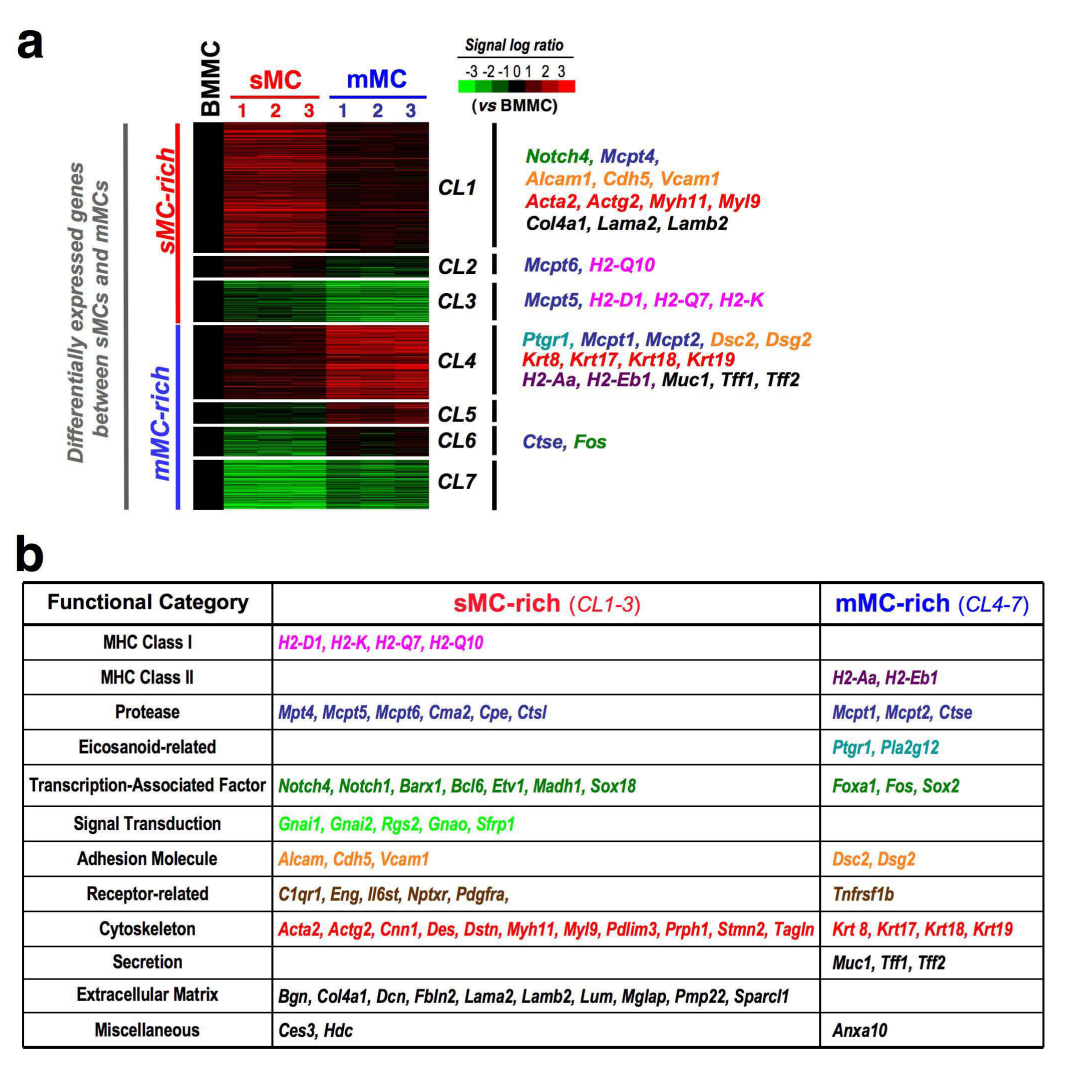
**Clustering analysis of the gene expression profiles between sMCs and mMCs**. (**a**) Representation of mRNA expression levels of sMC_1–3 _and mMC_1–3 _compared with BMMCs. The color of the bars represents the ratio of signal intensity between independent samples and BMMCs, according to the scale shown on the *top right*. Genes with significantly different expression between sMCs and mMCs (*p *< 0.05, Limma's *t *test) were selected (1,272 genes) and classified into 7 clusters using the *k*-means algorithm (*CL1-7*). (**b**) Functional categorization of representative genes from (a).

Of the ~12,000 genes represented on the oligonucleotide array, we selected 1,272 genes whose expression levels between sMC_1–3 _and mMC_1–3 _were significantly different (*p *< 0.05, Limma's *t *test). The expression level of each gene was normalized by its level in BMMCs, which are cultured MCs with so-called 'immature' properties, and the selected genes were classified into seven clusters using the *k*-means clustering algorithm (CL1-7; Figure 5a and Additional file 1). We also classified the genes into functional categories, and the representative genes are listed (Figure 5b). Among them, 666 genes (52.4%) showed sMC-biased expression (*CL1-3*); in 78% (519 genes) of sMC-rich genes, the expression levels were relatively low in BMMCs and augmented in sMC (*CL1&2*). For example, the expression level of *Mcpt4 *was relatively low in BMMCs, and if the expression profile of BMMCs reflects the immature properties of MC progenitors, *Mcpt4 *can be concluded to be induced during the final maturation into sMCs. Interestingly, the sMC marker genes *Mcpt5 *and *Mcpt6 *were classified into *CL2/3*, suggesting that these genes were expressed to some extent in 'immature' BMMCs, but their expression was suppressed during maturation into mMCs. On the other hand, 606 genes (47.6%) showed mMC-biased expression (*CL4-7*); in 51% (334 genes) of mMC-rich genes, their expression levels in BMMCs were low but were augmented in mMCs (*CL4&5*). For example, expression of *Mcpt1 *was low in 'immature' BMMCs but was drastically induced during maturation into mMCs.

### Protein expression of Notch4 in sMCs and Ptgr1 in mMCs in stomach tissue

**Figure 6:**
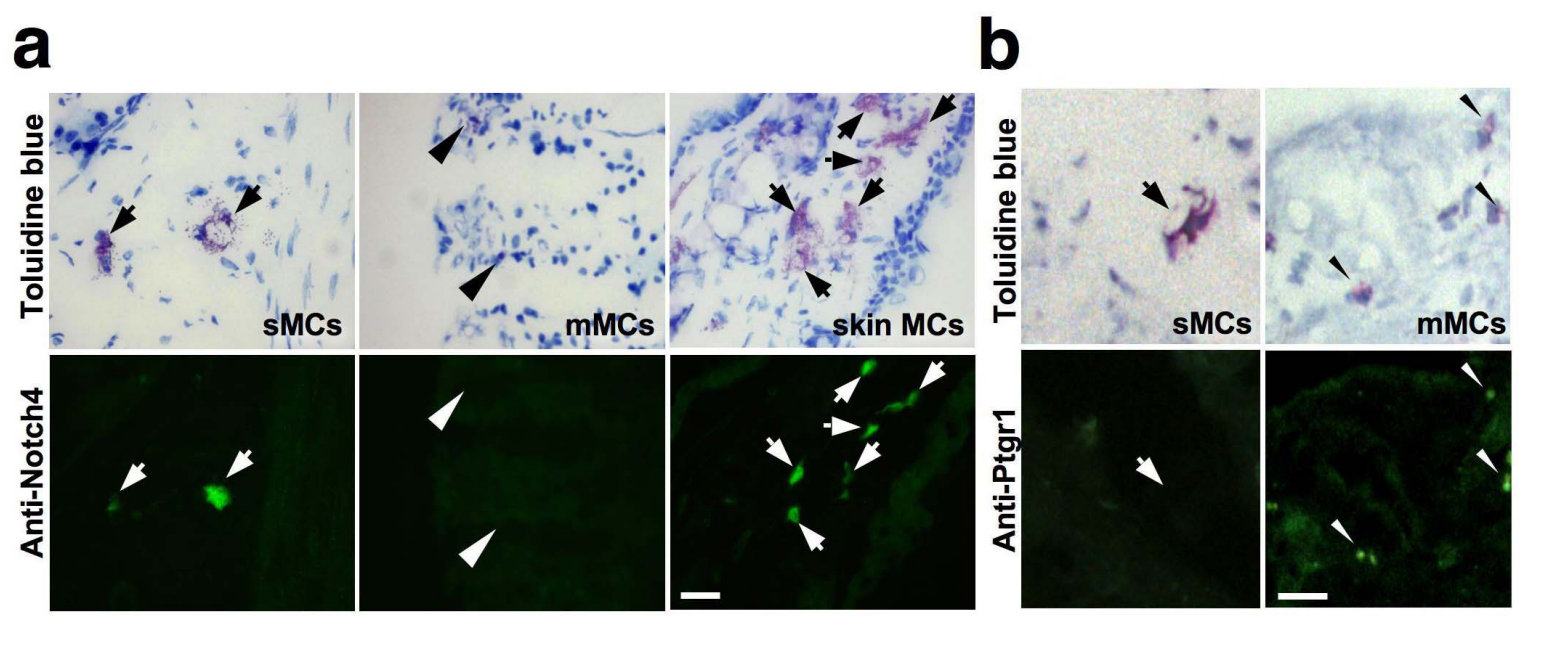
**Immunohistochemical analysis of Notch4 and Ptgr1 in sMCs and mMCs in stomach tissue**. (**a**) Stomach submucosa (sMCs; *left panels*), stomach mucosa (mMCs; *middle panels*) and skin (skin MCs; *right panels*) sections were stained with an anti-Notch4 antibody (*lower panels*) and with toluidine blue (*upper panels*). sMCs stained with the anti-Notch4 antibody in the gastric submucosa and skin dermis are indicated by arrows. No staining was observed in mMCs (*arrowheads*) localized in the gastric mucosa. sMCs and mMCs were metachromatically stained with toluidine blue. (**b**) Stomach submucosa (sMCs; *left panels*) and stomach mucosa (mMCs; *right panels*) sections were stained with an anti-Ptgr1 antibody (*lower panels*) and with toluidine blue (*upper panels*). No staining with the anti-Ptgr1 antibody was found in the sMCs (*arrow*). Small signals were observed in the mMCs (*arrowheads*). sMCs and mMCs were metachromatically stained with toluidine blue. *Bars*, 25 μm (a, b).

Among the genes showing differential expression (Figure 5b), we further focused on the expression of *Notch4 *in sMCs and *Ptgr1 *in mMCs, both of which have never been previously characterized in MCs. The *Notch4 *gene product is a member of the Notch family, consisting of transmembrane receptors which are activated by cell surface ligands on adjacent cells. Recent studies have suggested that Notch signaling is involved in lymphocyte and mast cell differentiation [30,31]. We first confirmed that *Notch4 *expression is significantly higher in the separately pooled sMCs than mMCs by real-time RT-PCR (data not shown). We next investigated whether the Notch4 protein is exclusively present in sMCs by immunostaining of stomach tissue (Figure 6a). Notch4 signals were detected in the nucleus-like structures of sMCs but not in those of mMCs. Furthermore, Notch4 signals were also found in the skin MCs, which were adjacently clustered with sMCs (Figure 3a). These results show that Notch4 is present in sMCs but not in mMCs, and suggest that Notch4 participates in sMC-specific transcription of Notch-target genes, which may be required for some sMC functions. In hematopoietic cells, it has been reported that constitutively active Notch4 promotes the expansion of progenitor cells and inhibits myeloid differentiation [32]. Since Notch ligands have been shown to exist in connective tissues such as skin dermis [33], it will be interesting to explore whether Notch4 plays a role in the differentiation of sMCs and the maintenance of sMC functions.

The *Ptgr1 *product, 15-oxo-prostaglandin 13-reductase/leukotriene (LT) B_4 _12-hydroxydehydrogenase is an essential enzyme for inactivation of eicosanoids such as prostaglandin E_2 _(PGE_2_) and LTB_4 _[34]. Although it has been reported that the pathways of eicosanoid synthesis differ among the different MC subclasses [1,4], our results suggest that the inactivation system of eicosanoids also varies among the MC subclasses. *Ptgr1 *expression was found to be significantly higher in the separately pooled mMCs by real-time RT-PCR (data not shown). We also examined Ptgr1 expression in stomach sections by immunostaining. Signals for the Ptgr1 protein were found in granule-like structures of mMCs in the stomach mucosa but not in sMCs (Figure 6b), suggesting that the Ptgr1 enzyme may be released from mMCs upon degranulation. Since PGE_2 _plays critical roles in the maintenance of gut homeostasis through mucosal protection and inhibition of acid secretion, it is possible that when activated, mMCs negatively regulate the cytoprotective actions of PGE_2 _through rapid inactivation by Ptgr1.

### Gene expression pattern of extracellular matrix components, adhesion molecules, and cytoskeletal proteins in sMCs and mMCs

MC phenotypes have been shown to depend on their interactions with the surrounding extracellular matrices (ECMs) and neighboring cells [1]. One of the most remarkable findings in this study is the difference in gene expression of ECM protein components, adhesion molecules, and cytoskeletal proteins, which may reflect functional adaptation of each type of MC to the mucosal or submucosal environment in the stomach (Figure 5b). mMCs express genes for mucosa-specific ECM proteins such as *Muc1 *(Mucin) and *Tff1 *(Trefoil factor), while sMCs express genes for conventional ECM proteins such as *Col4a *(procollagen) and *Lama2 *(laminin). Moreover, sMCs express genes for adhesion molecules such as *Alcam *and *Vcam1*, and genes for ordinary cytoskeletal proteins such as *Acta2 *(actin), while mMCs express desmosome-component genes such as *Dsc2 *(desmocollin) and *Dsg2 *(desmoglein), and genes for keratin intermediate filaments such as *Krt8 *and *Krt19*. Desmosomes were reported to be present in the stomach epithelia [35], and it was found that desmosome-like structures are detected in a particular type of MC [36]. It is thus possible that mMCs interact with adjacent epithelia through desmosomal adhesion in the stomach. In contrast, sMCs appear to interact with neighboring cells via adhesion molecules such as VCAM-1, ALCAM and VE-cadherin (*Vcam1*, *Alcam1 *and *Cdh5*). Since these adhesion molecules have been shown to be involved in dynamic regulation of the actin cytoskeleton [37,38], such molecule-mediated interactions with submucosal cells may be critical to maintain the functional and morphological properties of sMCs. Indeed, it should be noted that most sMCs are variable in shape, and are often stretched and winding as compared with mMCs [1].

## Conclusion

We established a method of RNA amplification from pooled intact MCs isolated from frozen tissue sections, which enables us to conveniently obtain the global gene expression pattern of MCs from various tissues, organs, and species including humans. By using this method, we demonstrated for the first time the distinct gene expression profiles of submucosal and mucosal MCs in the mouse stomach. Our findings offer insight into possible unidentified properties specific for each MC subclass.

## Methods

### Materials

The following materials were obtained from the sources indicated: HPLC purified T7-(dT)_24 _primer [5'-GGCCAGTGAATTGTAATACGACTCACTATAGGGAGGC GG(T)_24_] from GE Healthcare UK Ltd. (Buckinghamshire, England), RNase-free water, dNTP, SusperScript II, *Escherichia coli *(*E. coli*) RNase H, *E. coli *DNA polymerase I, *E. coli *DNA ligase, T4 DNA polymerase and random hexamers from Invitrogen (San Diego, CA), RNase inhibitor, glycogen, and MEGAscript T7 kit from Ambion (Austin, TX). Balb/c mice were obtained from JapanClea (Hamamatsu, Japan). This study was approved by the Committee on Animal Research of Kyoto University Graduate School of Pharmaceutical Sciences.

### RNA amplification and oligonucleotide microarray

Mouse interleukin-3-dependent BMMCs were prepared as described previously [39]. Total RNA of BMMCs was extracted using RNeasy mini kit (Qiagen, Valencia, CA). Five micrograms of total RNA from BMMCs were labeled and prepared for hybridization according to the manufacturer's instructions (standard protocol). On the other hand, 30 pg, 10 pg and 2 pg of BMMC total RNA were amplified and labeled by our original three-round amplification method, which is described below.

#### First round

Total RNA was incubated with T7-(dT)_24 _primer and first-strand cDNA was then synthesized by SuperScript II (Invitrogen). Second-strand synthesis was carried out by adding RNase H, DNA polymerase I and DNA ligase. The antisense RNA was synthesized using MEGAscript T7 kit.

#### Second round

The antisense RNA product was annealed with random hexamers, and cDNA was again synthesized by SuperScript II. Then, the RNA-cDNA hybrid was digested with RNase H and annealed with the T7-(dT)_24 _primer, and then second-strand synthesis was carried out by adding DNA polymerase I. The antisense RNA was again synthesized using MEGAscript T7 kit. Quality and size distribution of the antisense RNA product were confirmed by an RNA 6000 Nano LabChip on the Agilent Bioanalyzer (Palo Alto, CA).

#### Third round

As in the case of the second round, the double-stranded cDNA with a T7-promoter sequence was prepared from the second-round RNA product. Biotin-labeled antisense RNA was synthesized by RNA Transcript Labeling Kit (Enzo, Farmingdale, NY).

These labeled RNAs were hybridized to GeneChip Murine Expression oligonucleotide arrays (Affymetrix, Santa Clara, CA). We used microarray suite 5.0 of Affymetrix GeneChip Operating Software for quantification of the GeneChip data and decision of "Presence" or "Absence" of expression of each probe set using the values of 11 paired (perfect-matched and mismatched) probes.

### Microdissection of MCs from tissue sections, RNA extraction, and microarray data analysis

Tissue sections 7 μm in thickness were prepared using a Jung Frigocut 3000E cryostat (Leica, Nussloch, Germany), and thaw-mounted onto poly-L-lysine-coated glass slides. To visualize MCs, the sections were fixed with carnoy's fixative, and immersed in toluidine blue using the following protocol: carnoy's fixative for 1 min, RNase-free water for 10 sec, toluidine blue (0.5% in 0.12N hydrochloric acid) for 5 sec, RNase-free water for 10 sec, 70% ethanol for 15 sec, and 100% ethanol for 15 sec three times; the sections were then vacuumed for 10 min to dry. Each single MC was microdissected from the sections using a patch pipette, and 15 cells were collected with an LCM Cap using the PixCell Ile Laser Capture Microdissection System (Arcturus, Mountain View, CA). As a negative control, LCM Caps just put on tissue sections without MCs were subjected to the same protocols (no cell). Fifteen microdissected MCs were homogenized in denaturing buffer of RNeasy mini kits. Twenty nanograms of poly G (Sigma, Saint Louis, MO) was added to the lysate as a nucleic acid carrier, and total RNA was extracted. Fifty picograms of BMMC total RNA (BMMC-amp) and total RNAs extracted from sMCs in the stomach submucosa, mMCs in the stomach mucosa and skin MCs in the ear dermis were amplified and labeled using the three-round amplification method, and were hybridized to U74Av2 Murine Genome Array (Affymetrix). On the other hand, total RNA of BMMCs (BMMC-std) and peritoneal MC, which were collected from mouse peritoneal cavities and purified by density gradient centrifugation using metrizamide, were labeled and hybridized by the standard protocol. Raw microarray data of macrophages (E-MEXP-38/298290452) and fibroblasts (E-GEOD-6697/1629511747) using the standard protocol were obtained from ArrayExpress, a public repository for transcriptomics data. We used either microarray suite 5.0 of Affymetrix GeneChip Operating Software or the robust multi-array average (RMA) expression measure for log transformation (log_2_) and normalization of the GeneChip data [40,41]. To determine the similarity in the data, hierarchical clustering analysis and PCA using the R statistical environment  were performed as a visualization technique. For comparison of the expression profiles of sMCs with that of mMCs, we selected 1,272 genes identified as having significantly different expression levels by the Limma's *t*-test (*p *< 0.05, *n *= 3). Signal values of sMCs and mMCs were normalized by the signal values of BMMCs. Using the *k*-means clustering algorithm, these genes were classified into seven clusters on the basis of similarity of their expression profiles.

### Real-time reverse-transcription polymerase chain reaction (RT-PCR)

Total RNA extracted from 60 captured MCs was subjected to real-time RT-PCR. Real-time PCR was performed in a LightCycler (Roche, Mannheim, Germany) using Fast Start DNA Master SYBR Green I. The expression level of each gene was quantified using external standardized dilution, and normalized by 28S ribosomal RNA. Primer sequences are shown in Table 2. The specificity of the primers was confirmed by checking the product size and restriction enzyme pattern by gel electrophoresis and the melting temperature (data not shown).

**Table 2:** List of primers used for real-time PCR analysis.

Gene Symbol	Forward primer (5' -> 3')	Reverse primer (5' -> 3')
*Kit*	ATAGACCCGACGCAAC	AATAAACGAGTCACGCT
*Fcer1a*	GCCCCGTCTCCATTAG	CAATAACCCCGTGTCC
*Mcpt1*	AAACAGTCATAAATGGCAAG	GGGAACAAACCATCATCAC
*Mcpt2*	TTCATTGCCTAGTTCCTCT	CTTTTCAGCTACTTGCTCT
*Mcpt4*	CCTTACATGGCCCATCT	CTTCCCCGGCTTGATA
*Cma2*	GCGGAAATGCAAAGCC	ACAGGGAACAGTCCATC
*Anxa10*	TACCCACAACTTCGGC	GGCAAGTAGTGCTTTCT
*Ctse*	GCAAGCCTATTGGCAG	TGGCATCGTGTCGAGA
*Fos*	TGTGTACTCCCGTGGT	ACGAACAGGTAAGGTCC
*Ptgr1*	CATCGTGAATCGGTGG	GCTAGGTCAAACGCAT
*Cnn1*	ACGGCCTACGGTACAC	GGTACTCCGGGTTCAG
*Ces3*	AGTGATTGTGTCTCGAAG	GTTCCCATTCCGAGCA
*Cpe*	ACCGGAAGAGACTCTCA	CCAGTAATCCCCATCCT
*Notch4*	CCCTTAAACTCGGTTGT	GGTGCTTAATAAATAGTTGCC
28S rRNA	CAGTACGAATACAGACCG	GGCAACAACACATCATCAG

### Immunostaining

For tissue staining, frozen sections were fixed in 4% formaldehyde and incubated with a rabbit anti-Notch4 antibody (1:20, Santa Cruz Biotechnology, Santa Cruz, CA) or a rabbit anti-Ptgr1 antibody (1:20) which was a kind gift from Prof. Takao Shimizu (University of Tokyo) [42].

## Abbreviations

BMMC: bone marrow-derived mast cell; CL: cluster; sMC: submucosa mast cell; DEPC: diethylpyrocarbonate; ECM: extracellular matrix; LCM: laser capture microdissection; LT: leukotriene; MC: mast cell; mMC: mucosa mast cell; PCA: principal component analysis; PG: prostaglandin; r: correlation coefficient; RMA: robust multi-array average; rRNA: ribosomal RNA; RT-PCR: reverse transcription-polymerase chain reaction.

## Authors' contributions

ST designed the research, performed the research and wrote the paper; YT performed the research and wrote the paper; ES-N wrote the paper; YO performed the microarray data and statistical analysis; ST performed the research; GT designed the research; ST performed the research; YS designed the research and wrote the paper. Conflict-of-interest: The authors declare no competing financial interests.

## Supplementary Material

Additional file 1 **Genes with significantly different expression between sMCs and mMCs.** The list represents 1,272 genes significantly altered between sMCs and mMCs in the order of clustering (Figure 5a). The values represent expression levels normalized to those of BMMCs. Click here for file
